# Wheat Grain Protein Content under Mediterranean Conditions Measured with Chlorophyll Meter

**DOI:** 10.3390/plants10020374

**Published:** 2021-02-15

**Authors:** Marta Aranguren, Ander Castellón, Ana Aizpurua

**Affiliations:** Department of Plant Production and Protection, NEIKER-Basque Institute for Agricultural Research and Development, Basque Research and Technology Alliance (BRTA), Parque Científico y Tecnológico de Bizkaia, P812, 48160 Derio, Biscay, Spain; maranguren@neiker.eus (M.A.); acastellon@neiker.eus (A.C.)

**Keywords:** *Triticum aestivum*, Precision Agriculture, Yara N-Tester^TM^, bread-making flour

## Abstract

Adequate N fertilisation is crucial to increase the grain protein content (GPC) values in wheat. The recommended level of GPC needed to achieve high-quality bread-making flour should be higher than 12.5%. However, it is difficult to ensure the GPC values that the crop will achieve because N in grain is derived from two different sources: N remobilized into the grain from N accumulated in the pre-anthesis period, and N absorbed from the soil in the post-anthesis period. This study aimed to (i) evaluate the effect of the application of N on the rate of stem elongation (GS30) when farmyard manures are applied as initial fertilisers on GPC and on the chlorophyll meter (CM) values at mid-anthesis (GS65), (ii) establish a relationship between the CM values at GS65 and GPC, and (iii) determine a minimum CM value at GS65 to obtain GPC values above 12.5%. Three field trials were performed in three consecutive growing seasons, and different N fertilisation doses were applied. Readings using the CM Yara N-Tester^TM^ were taken at GS65. The type of initial fertiliser did not affect the GPC and CM values. Generally, the greater the N application at GS30 is, the higher the GPC and CM values are. CM values can help to estimate GPC values only when yields are below 8000 kg ha^−1^. Additionally, CM values at GS65 should be higher than 700 to achieve high-quality bread-making flour (12.5%) at such yield levels. These results will allow farmers and cooperatives to make better decisions regarding late-nitrogen fertilisation and wheat sales.

## 1. Introduction

In order to feed a growing population, it is imperative to have increasing wheat grain yields and high grain quality. Grain quality is characterized in different ways, such as hardness, specific weight, Chopin Alveograph, Zeleny volume, and Hagberg number [1], but the main indicator is the grain protein content (GPC, [2]). Grain protein content prediction is complex because it depends on several aspects related to crop nitrogen (N) utilization, such as genetics (variety), environmental factors, and agronomic management practices such as N fertiliser application [3]. N fertilisation is a crucial factor for increasing yields and GPC. The amount of N applied to wheat must be carefully managed to the balance yield, grain quality, and environment needs, adjusting the N supply and crop requirements [4]. However, it is difficult to increase grain yield and quality concurrently due to its negative relationship [5].

In the area where this study was located (Araba, Basque Country, northern Spain), low GPC values have been reported due to the high yields achieved [2]. Additionally, the varieties commonly used have been selected to obtain high yields rather than obtaining high GPC. In this area, the beginning of the tillering (GS21, [6]) and stem elongation (GS30, [6]) growing stages are clues to establishing the required N fertilisation. The usual application rate is 40–60 kg N·ha^−1^ at GS21, and a greater but variable application at GS30. In some cases, a third late N fertiliser application at leaf-flag emergence (GS37, [6]) has also been considered to increase the grain N concentration because weather conditions are humid around the fertilisation moment (beginning of May in our conditions; [2]). 

The N utilization of crops involves several processes, such as N uptake, assimilation, translocation, and remobilization [7]. Grain N is derived from two different N sources: N that is absorbed in the post-anthesis period from the soil (GS60–GS90; [6]) and N remobilized to the grain that was accumulated in vegetative organs in the pre-anthesis period (until GS60). After anthesis, vegetative organs behave as N sources, protein hydrolysis occurs, and amino acids are transported to the grain [7]. Thus, a large proportion of N in grain (60–95%) might come from N remobilized rather than being taken from the soil [1], but it might depend on the weather conditions [2]. Thus, because the grain-filling process depends on several factors, the in-season assessment of GPC remains challenging in cereals. The recommended level of GPC for high-quality bread-making flour should be higher than 12.5% [8]. Farmers who grow cereals to achieve high-quality grain cannot predict if the crop will have the required protein standard [1]. The main concern is to ensure the GPC values that the crop will achieve and determine in advance whether an extra N rate is required. Not applying it when there is no need would benefit the farmer economically and would be environmentally friendly, avoiding N leaching and N gaseous losses [9]. Additionally, a better estimation of the GPC would help cooperatives improve the planning of their sales strategies because they could determine beforehand the percentage of grain that could be sold for bread-making flour or as animal feed.

Because a significant proportion of N in the grain has been remobilized from the leaves, stem, and roots of the plant in the post-anthesis period, it is reasonable to measure the N content from the shoot part at mid-anthesis (GS65, [6]) to predict GPC and anticipate whether a late N supply is necessary. Leaves are the most important organs in terms of N reserves, accounting for up to 50–62% of the total N of the plant [7]. The leaf flag was shown as a good indicator of the whole shoot N status at GS65 [1], and has been used to predict GPC in winter wheat [10]. Measuring N in the plant leaves is not practical because it requires destructive and time-consuming procedures such as sampling and laboratory analysis, making it impractical for farmers. In this sense, it is necessary to have rapid results and easy measurements of the shoot N status to develop reference values to decide whether a late N supply is necessary and predict the GPC at harvest. 

Chlorophyll meters such as Minolta SPAD (Minolta corporation, Ltd., Osaka, Japan) or Yara N-Tester^TM^ (Yara International ASA, Oslo, Norway) can provide instantaneous results for diagnostic purposes. Arregui et al. [11] and Ortuzar-Iragorri et al. [12] found good relationships between chlorophyll meters and N content of plant leaves in the same climatic conditions of the present field experiment, making them interesting to obtain rapid results. Relationships between remote sensing tool measurements against GPC have been studied in cereals, but there were no consistent results across locations and years [10,13,14]. In a previous study, Aranguren et al. [15] showed no general relationship across years between RapidScan CS-45 (NDVI (Normalized Difference Vegetation Index) and NDRE (Normalized Difference Red Edge)) or Yara N-Tester^TM^ and GPC values under humid Mediterranean conditions. However, they detected that the relationship between the absolute chlorophyll meter values at GS65 (mid-anthesis; [6]) and GPC across years (*R*^2^ = 0.35) was improved compared with NDVI and NDRE [15]. They attributed that low predictability to the mentioned lack of consistency across years, as other authors have shown [1]. As previously mentioned, several variables, such as yield, the moment of crop N absorption, or remobilisation efficiency, might affect the grain filling process [13]. Therefore, based on previous results [15] and literature review [1,9,16], it was hypothesized that chlorophyll meter readings might be helpful in understanding GPC in wheat, but is necessary to study and understand the effect of other variables affecting the grain-filling process.

This study performed under humid Mediterranean conditions was aimed to (i) evaluate the effect of the application of a variable N rate at stem elongation (GS30) when farmyard manure is applied as initial fertilisers to chlorophyll meter values at mid-anthesis (GS65) and GPC values, (ii) determine the possibility of establishing a relationship between the chlorophyll meter values at GS65 and GPC, and (iii) establish the minimum chlorophyll meter value needed at GS65 to obtain GPC values above 12.5%. 

## 2. Results

The interaction among the growing season, initial fertilisation treatment, and N rate at GS30 was significant. Therefore, each factor was analysed depending on the other factors. The differences among the growing seasons are not presented in Table 1 and Table 2 because of the high volume of data; thus, they are presented in supplementary Tables S1 and S2. However, if differences were mentioned in the main text, they were considered statistically significant.

### 2.1. Wheat Grain Protein Content (GPC) and Yield

The GPC varied from 7.4% to 10.9% in 2015, from 7.5 to 10% in 2016, and from 8.4 to 13.3% in 2017 (Table 1). In 2017, the GPC values were generally higher and the range of variation was wider than those in 2016 and 2015 (Table S1). In 2016, no differences were detected in the GPC values among the treatments (Table 1). However, in 2015 and 2017, significant differences were detected in the GPC values derived from the N rate applied at GS30, but not from the initial fertilisation treatments. In 2015, the highest GPC values were achieved with the 120 kg N·ha^−1^ N rate in conventional and dairy slurry treatments; however, in sheep manure, the highest GPC values were achieved with 160 kg N·ha^−1^. In 2017, the GPC values increased together with the N rates applied at GS30. GPC values above 12.5% were only achieved in 2017 with the highest N rate applied at GS30 (160 kg N·ha^−1^). 

The amount of mineral N fertiliser at GS30 significantly influenced the yield values in the three growing seasons. The wheat grain yield varied between 4300 and 8700 kg·ha^−1^ in 2015, 6000 and 10800 kg·ha^−1^ in 2016, and 3400 and 7000 kg·ha^−1^ in 2017 (Table 1). The maximum wheat grain yields were achieved with 80 kg N·ha^−1^ applied at GS30 in 2015 under all the initial fertilisation treatments (conventional, dairy slurry, and sheep manure) and in 2016 and 2017 under conventional and dairy slurry initial fertilisation treatments. The maximum wheat grain yields were achieved with 120 kg N·ha^−1^ applied at GS30 in 2016 and 2017 with sheep manure treatments.

### 2.2. Yara N-Tester^TM^ Readings and GPC Prediction

At the GS65 growing stage, the lowest Yara N-Tester^TM^ values were 470, 450, and 460 for 2015, 2016, and 2017, respectively, and the highest values were 660, 610, and 710 for the same years, respectively (Table 1). In 2015, with conventional treatment, the highest Yara N-Tester^TM^ values were achieved with the 80 kg N·ha^−1^ rate applied at GS30, with dairy slurry treatment with the 160 kg N ha^−1^ rate, and with sheep manure at the 120 kg N·ha^−1^ rate. In 2016, with conventional treatment, no differences were found in the Yara N-Tester^TM^ readings. In 2016, with the dairy slurry treatment, the maximum Yara N-Tester^TM^ values were achieved with the 80 kg N·ha^−1^ rate applied at GS30, and with sheep manure with the 120 kg N ha^−1^ rate. In 2017, (Table 1), under the three initial fertilisation treatments, the Yara N-Tester^TM^ maximum values were achieved with the 120-kg N·ha^−1^ rate. 

**Figure 1:**
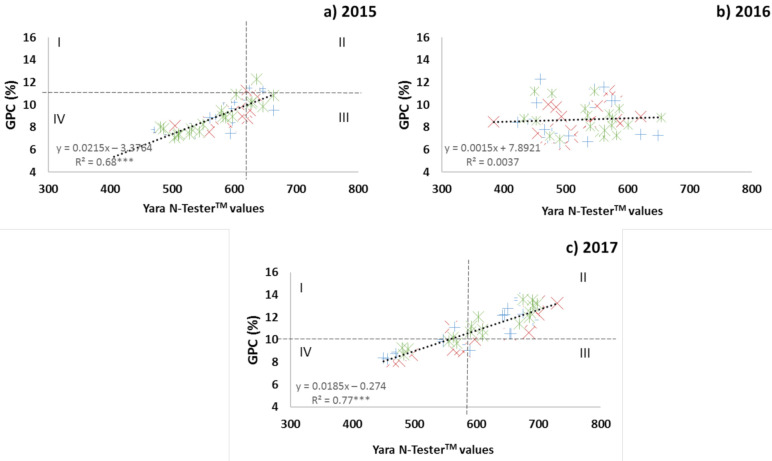
Relationship between the Yara N-TesterTM values at GS65 and% GPC in the 2015 (**a**), 2016 (**b**), and 2017 (**c**) growing seasons at Arkaute. Type of initial fertilisation: Conventional +; DS: Dairy slurry x; SM: Sheep manure *. The linear model was fitted. ***, significant at the 0.001 probability level. Strip lines indicate the critical x value and critical y value separating the data into four quadrants following the Cate–Nelson procedure. The Roman numerals indicate the quadrant of the plot. The data inside quadrants II and IV are concordant with the regression. The data inside quadrants I and III are not in concordance.

In 2016, the Yara N-Tester^TM^ values at GS65 could not explain the GPC variability (Figure 1b). However, in 2015 and 2017, the Yara N-Tester^TM^ values at GS65 could explain 68% and 77% of the GPC variability, respectively (Figure 1a,c). The Cate–Nelson procedure can identify the critical level that best divides the data into two populations. The points in quadrant II represent the population with the highest GPC values, and those in quadrant IV the population with the lowest GPC values. The critical level is that at which *R*^2^ reaches a maximum. The Yara N-Tester^TM^ critical value dividing the two populations was 619 in 2015 and 591 in 2017. The GPC critical value obtained was 11.23 in 2015 and 10.29 in 2017. In 2015, the number of points in quadrant II was much lower than that in quadrant IV. In 2017, the dispersion of the points in the relationship was uniform in quadrants II and IV. The points located in quadrants I and III are considered outliers and correspond to the overestimation or underestimation of GPC, respectively. The error of the Yara N-Tester^TM^, calculated as the percentage of outliers in relation to the total of points, was 28% in 2015 and 6% in 2017. In 2015, there were more error points mainly located in quadrant III (underestimation) and corresponding to yields higher than 8000 kg ha^−1^. Thus, the Yara N-Tester^TM^ and GPC values differ in their relationship when the yields are lower or higher than 8000 kg ha^−1^. When the yields are lower than 8000 kg ha^−1^, the relationship between the Yara N-Tester^TM^ values and GPC values increase linearly; when the yields are higher than 8000 kg ha^−1^, the relationship is unclear. 

**Figure 2:**
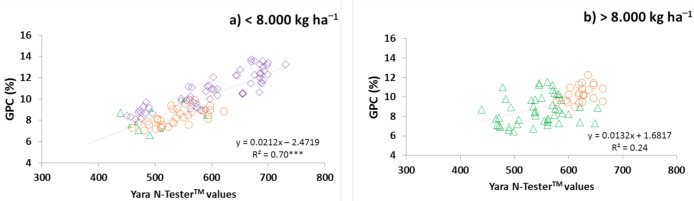
Relationship between the Yara N-Tester^TM^ values at GS65 (mid-anthesis; Zadoks et al., 1974) and GPC (%) when the wheat grain yields are lower than 8000 kg N ha^−1^ (**a**) and when the wheat grain yields are higher than 8000 kg ha^−1^ (**b**) in the field study at Arkaute. Wheat growing seasons: 2015**◯**; 2016**∆**; 2017**◊**. The linear model was fitted. ***, significant at the 0.001 probability level. Strip lines indicate the critical × value and critical y value separating the data into four quadrants following the Cate–Nelson procedure. The Roman numerals indicate the quadrant of the plot. The data inside quadrants II and IV are concordant with the regression. The data inside quadrants I and III are not in concordance.

Therefore, the coefficients of determination (*R*^2^) for the relationships between Yara N-Tester^TM^ values at GS65 and GPC were calculated based on the 8000 kg ha^−1^ yield. When the yields were lower than 8000 kg ha^−1^, the capacity to predict the GPC variability from Yara N-Tester^TM^ readings was similar among the three growing seasons (*R*^2^ = 0.75; Figure 2a). Yields lower than 8000 kg ha^−1^ in 2015 occurred in the three lowest N rates (0, 40, and 80 at GS30) under DS and SM treatment and in the two lowest rates (0 and 40 at GS30) under conventional treatment. In 2016, yields lower than 8000 kg ha^−1^ occurred with X + 0N treatment, and in 2017 with all treatments. The error of Yara N-tester^TM^ calculated as the percentage of outliers in relation to the total of points was 5%. When the yields were higher than 8000 kg ha^−1^, no significant relationship was found between GPC and Yara N-tester^TM^ readings at GS65 (Figure 2b). 

### 2.3. Factors That Might Affect the GPC Predictability

Regarding the grain total N at harvest (Table 2 and Table S2), in 2016, the values were generally higher (63–168 kg N ha^−1^) than those in 2015 (51–149 kg N ha^−1^) and 2017 (46–146 kg N ha^−1^). The higher the N rate is at GS30, the higher the grain total N values in the three growing seasons. In 2015 and 2016, the maximum values were achieved with the 120 kg N ha^−1^ rate applied at GS30; however, in 2017, the maximum value was achieved with the highest N rate (160 kg N ha^−1^). In 2016, the DS + 0N and SM + 0N treatments presented higher values than the 40N + 0N treatment.

No significant differences among the N treatments were detected in the post-anthesis N increase in the aerial part of the wheat crop from GS65 to harvest in any of the growing seasons (Table 2). However, treatments in the 2016 growing season presented a slightly higher N increase during the post-anthesis period (24–59 kg N ha^−1^) than treatments in 2015 (6–32 kg N ha^−1^) and 2017 (11–37 kg N ha^−1^; Table S2). The differences between conventional treatment and treatments with organics as initial fertilisers without N application at GS30 (0 kg N ha^−1^) were only significant in 2017. In that case, the conventional treatments achieved higher values (30 kg N ha^−1^) than the treatments with organics as initial fertilisers (13–19 kg N ha^−1^). 

## 3. Discussion

### 3.1. Periods Affecting Grain Protein Content (GPC) Prediction in Wheat

The N accumulated at pre-anthesis, mid-anthesis (GS65), and post-anthesis may affect N partitioning at the plant level [17] and, thus, the GPC values in wheat crop. In the present study, each kg of N applied at GS30 (pre-anthesis period) had a different effect depending on the growing season. Although grain total N was similar in 2015 and 2017 (Table 2), the GPC values were higher in 2017 than in 2015 (Table 1). In 2017, rain did not alter the N application at GS30, and this dry period persisted until GS37 (Table S3), causing a late N uptake (from GS37 onwards), low yields (3,800–7000), and higher GPC values (8.4–13.3%). In the 2017 growing season, the NDVI values started increasing from GS37, and biomass accumulation (sink size) was low from GS30 to GS37 (data not shown). López-Bellido et al. [1] reported that high values of the grain N concentration and low yields were obtained in the years with dry conditions, such as 2017 (Table S3). Additionally, some authors have reported significant increases in the GPC in wheat in the same edaphoclimatic area when N is applied after GS37 [2,9]. In relation to the 2016 growing season, the grain total N was higher than that in the other two growing seasons (Table 2). In 2016, the N applied at GS30 had a huge effect on the yield because, as the N rate applied at GS30 increased, the yield values increased more than that in the other two growing seasons. By contrast, the GPC values did not increase with the increases in the N rate applied at GS30 (Table 1). The grain total N values, high yields, and achieved GPC values showed a clear dilution effect. In 2016, the NDVI values were high from GS30 to GS65 (data not shown). Thus, the crop sink capacity was high since early in the growing season. Bogard et al. [18] showed that an increased crop sink capacity strongly affects the grain yield. The issue is that the grain yield and grain quality are difficult to improve simultaneously due to the negative relationship between them [5]. Increasing the yield might have an important effect on reducing the GPC due to the dilution effect of carbon-based compounds [1,19,20]. Fuertes-Mendizábal et al. [2] mentioned that climatic conditions in Araba could lead to very high yields, and therefore, to a low GPC due to the dilution effect. A drawback of the remarkable increase in grain yield obtained through breeding in wheat has been the decreased GPC [21,22].

Yara N-Tester^TM^ readings taken at mid-anthesis (GS65) in the leaf flag could explain the GPC variability in the 2015 (Figure 1a) and 2017 (Figure 1c) growing seasons (*R*^2^ = 68% and 77%, respectively). That correlation was reasonable because the flag-leaf provides N more directly to the spike, and a strong depletion of more than 50% occurs in the flag-leaf N content during the grain-filling period [23]. However, the relationship between the chlorophyll meter readings and GPC variability was growing season dependent. Thus, in 2016, it was not possible to explain the GPC variability from the Yara N-Tester^TM^ readings (Figure 1b). The Cate–Nelson test showed that the critical values in 2015 divided the population into two different subpopulations (one with higher GPC values, and the other with lower GPC values), whereas the Yara N-Tester^TM^ and GPC values differed in their relationship. That division matched with yields of approximately 8000 kg·ha^−1^. When the yields exceeded 8000 kg·ha^−1^ (Figure 2b), no clear pattern in the relationship was observed between Yara N-Tester^TM^ values and GPC values, similar to the findings for most of the treatments in 2016 (except for the treatments with 0 kg N·ha^−1^ applied at GS30) and treatments with the highest N rates in 2015. De Oliveira Silva et al. [24] mentioned different hypotheses to explain the opposite relationship between grain yield and protein, such as dilution of protein in higher yield, competition for energy and assimilates between biomass and N during grain formation or the different accumulation rates between grain protein and carbohydrates during grain filling period. However, when the yields were lower than 8000 kg·ha^−1^, the GPC variability prediction capacity from Yara N-Tester^TM^ readings was similar in the three growing seasons (*R*^2^ = 0.75; Figure 2a). Other authors, such as Turley et al. [25] with yields ranging from 3800 to 8500 kg·ha^−1^ and López-Bellido et al. [1] with yields ranging from 4000 to 11,000 kg ha^−1^, showed that taking chlorophyll meter readings at the leaf flag from GS60 to GS69 was adequate for GPC variability prediction. They remarked that, when the yields were high, chlorophyll meter readings presented higher variability than when the yields were lower. Le Bail et al. [26] suggested that, together with the chlorophyll meter readings, the ear number per square metre should be measured to obtain a measurement related to the yield. Models to predict wheat yields have been developed [27,28] that allow the determination of the yields in advance and predict how the yield would affect the GPC values.

In 2016, the post-anthesis N increase in the aerial part was higher than that in the other two growing seasons (Table 2), likely because of the higher amount of biomass accumulated throughout the growing season, justifying the high yield values. Additionally, Mi et al. [29] reported that a high sink size promotes the post-anthesis N uptake to meet the crop N requirements. The N uptake in the post-anthesis period can contribute from 5 to 50% of the grain N, but it depends on the environmental conditions [7]. Giunta et al. [21] mentioned that rainfall during post-anthesis in particular was the main driver of grain yield and GPC, either directly or via its interaction with nitrogen availability. The mean rainfall of the area in the period between GS65 to harvest (1981–2010) was 81 mm [30]. In this study, wet conditions occurred in the three growing seasons after mid-anthesis (Table S3), with the total rainfall being 56 mm in 2015, 71 mm in 2016, and 114 mm in 2017. The number of days elapsed (Table S3) in 2016 from GS65 to harvest was more (69) than that in 2017 (63) and 2015 (54). In 2016, the soil moisture was higher, and the crop had more time to absorb N than in the other two growing seasons. The higher uptake capacity and the more time elapsed in the post-anthesis period might explain the higher post-anthesis N increase. The effect of the post-anthesis N increase on the variability of the GPC values in winter wheat was also described by Monaghan et al. [17]. They concluded that the significance of the post-anthesis N absorbance in the GPC variability is related to the different N partitioning accumulated before and after anthesis. The N absorbed after anthesis is more efficiently destined to the grain because the N absorbed after anthesis does not include the crop growth response [18]. However, it is very complex to predict how much N will be absorbed by the crop in the post-anthesis period [14]. The GPC value range was similar in 2015 and 2016, although the chlorophyll meter readings were lower in 2016 than in 2015. Another reason that could make the GPC variability prediction difficult is predicting how efficiently the plant will translocate N into the grain in the grain-filling period (GS60–GS90) [14]. In the present experiment, the amount of N translocated to grains from the crop total N was 80% or higher (data not shown). Fuertes-Mendizábal et al. [23], using the same wheat variety and under the same edaphoclimatic conditions, found that the NHI (Nitrogen Harvest Index) values ranged between 75 % and 82 %.

In summary, the chlorophyll meter readings can predict the GPC values when the yields are lower than 8000 kg N ha^−1^, but not when the yields are higher. The established sink size and post-anthesis conditions may affect that prediction, as in 2016. Some authors have mentioned that the GPC predictability from chlorophyll meters is better when the readings are taken at the beginning of the milk stage (GS71, [6]) than at the anthesis stage [26]. However, López-Bellido et al. [1] suggested that taking chlorophyll meter readings later in the growing season would improve the predictability, but leaf senescence (starting at medium milk; GS75, [6]) should be considered. Additionally, it would be too late to perform N application.

### 3.2. Minimum Chlorophyll Meter Readings to Achieve GPC Values of 12.5%

The recommended level of GPC needed for the necessary bread-making quality should be higher than 12.5% [8]. However, it is difficult to ensure that this value will be achieved because many factors influence the grain-filling process (GS60–GS90) [17]. As stated above, achieving the GPC needed for the necessary bread-making quality is complex under the humid Mediterranean conditions of Araba using the usual fertiliser practices (last N application at GS30). Additionally, the commonly used varieties, such as Cezanne variety, have been selected to obtain high yields rather than to obtain a high GPC. The 12.5% value was only achieved in 2017 with the highest N rate at GS30 (160 kg N·ha^−1^) in the three initial fertilisation treatments and with yields ranging from 6000 to 7000 kg ha^−1^. To enhance the GPC, it would be useful to determine the minimum Yara N-Tester^TM^ reading value to achieve the required bread-making quality. As mentioned above, predicting the GPC variability using Yara N-Tester^TM^ in this area under the humid Mediterranean climate was only possible when the yields were lower than 8000 kg·ha^−1^. Several authors have established minimum chlorophyll meter readings to achieve the GPC value of 12.5% [1,16,25]. Some of those studies used Minolta SPAD, which is a chlorophyll meter similar to Yara N-Tester^TM^. Both tools measure the light transmitted at 650 and 940 nm and supply different units in readings, but they are highly correlated as observed in our previous calibration experiments (NTester reading = 14.1 × SPADreading − 61.54; *R*^2^ = 0.90; data not shown). Arregui et al. [11], under the same climatic conditions, also found that both tools are also highly correlated with a very similar equation. Turley et al. [25] reported that, to achieve GPC values approximately 11% (with yields approximately 8000 kg ha^−1^), the Minolta SPAD reading values should be 48, which corresponds to a Yara N-Tester^TM^ value of 615 (calculated from the previously mentioned equation). In the present study, to achieve GPC values approximately 11%, the Yara N-Tester^TM^ reading values were similar (approximately 620; Figure 2a) to those proposed by Turley et al. [25]. López-Bellido et al. [1] showed that, to achieve GC values of 12.5% (with yields approximately 10,000 kg ha^−1^), the necessary Minolta SPAD reading values at GS65 should be 51 or higher, corresponding to a Yara N-Tester^TM^ value of 658 (calculated from the previously mentioned equation). In the present study, the Yara N-Tester^TM^ values to achieve a GPC of 12.5% were higher (approximately 700, Figure 2a) than those proposed by López-Bellido et al. [1]. Miller et al. [16] reported that, tor achieving GPC values of 13% (with yields ranging from 1100 to 8000 kg·ha^−1^), the necessary Minolta SPAD readings should be higher than 40, which corresponds to Yara N-Tester^TM^ = 503 (calculated from the previously mentioned equation), with the values lower than those required in the present study (approximately 720; Figure 2a). The variation in the results demonstrates that it is necessary to be cautious regarding the universality of chlorophyll meter values across geographical locations, as López-Bellido et al. [1] reported. Additionally, the wheat variety should also be considered because each variety should have its own calibration [4]. To generalize the relationship between the chlorophyll meter readings and GPC values, some authors have proposed the use of normalised values [4], which are calculated as a percentage by assessing 100% to a non-limiting area of the field. However, data normalisation has limitations because finding a control strip that is representative of the entire field is challenging. Ravier et al. [31] showed that it is not easy to ensure that an overfertilised fringe is not N deficient, thereby complicating the use of normalised data. Additionally, using normalised values makes the use of chlorophyll meters more complicated for farmers. In the present study, the GPC predictability was the worst using Yara N-Tester^TM^ normalised values (data not shown). Hoel [32] reported that correction factors (as +10 or −10, or +20 or −20) could be used to normalise the differences among varieties when absolute chlorophyll meter readings are used. Hoel [32] reported that, with some varieties, it was not necessary to use a correction value because they presented similar leaf greenness.

## 4. Materials and Methods

### 4.1. Study Site

Three field trials were established in Arkaute (Araba, Basque Country, Spain) at NEIKER facilities in three consecutive wheat growing seasons defined as 2015, 2016, and 2017 in different fields under rainfed conditions. The three field trials were flat, presented similar characteristics, and the distance among them was 130 m. Other soil properties were described previously [33].

### 4.2. Climate

The climate of the area was humid Mediterranean according to the water regime of Papadakis’ [34] classification. The total rainfall (mm) and days elapsed between relevant key wheat growing stages in the three growing seasons are shown in Table S3. 

### 4.3. Experimental Setup and Treatments

The experiment was a factorial randomized complete block design with three factors (year, initial fertilisation, and N rate at stem elongation) and four replicates. Three kinds of initial fertilization were applied: Dairy slurry (40 t·ha^−1^), sheep farmyard manure (40 t·ha^−1^), and conventional treatment (no organic fertilizer basal dressing and 40 kg N·ha^−1^ at tillering). These three types of fertilization were combined with five N rates (calcium ammonium nitrate, NAC 27%) in the topdressings applied at GS30 (0, 40, 80, 120, and 160 kg N·ha^−1^). Apart from the treatments, two controls were established (Table 3): A control without N fertilisation (0 N), and an overfertilised control plot (280 N). Information regarding the organic manure characteristics and application was previously described [33]. Soft wheat (*Triticum aestivum* var. Cezanne) was sown at a 220 kg seed·ha^−1^ rate on 24−11-2014, 06-11-2015, and 18-11-2016, and was harvested on 21-07-2015, 2-08-2016, and 2-08-2017.

### 4.4. Yield and GPC 

Grain yields were harvested at crop maturity using a plot harvester (1.5 m × 8 m) and were converted to a 12% dry matter basis. The grain samples were oven-dried at 70 °C for at least 48 hours and then ground through a 1-mm screen before the determination of the total N concentration using the Kjeldhal procedure [35]. GPC was determined as the product of the grain N concentration multiplied by 5.7 [36]. 

### 4.5. Increase in the N Content in the Aerial Part during the Post-anthesis Period (from GS65 to Harvest)

N accumulation in the aerial part of the wheat crop during the post-anthesis period (from GS65 to harvest) was measured in all conventional treatments, DS + 0N, SM + 0N, and 0N (Table 3), and was calculated as follows:

(1) Post-anthesis N increase (kg N·ha^−1^) = Crop Total N – GS65 Total N (1)
(2) Crop Total N content was calculated as:
  Crop Total N (kg N·ha^−1^) = Grain Total N (kg N·ha^−1^) + Straw Total N (kg N·ha^−1^) (2)
  where Grain Total N content was calculated as:
(3) Grain Total N (kg N·ha^−1^) = Grain Yield (kg·ha^−1^) × Grain N concentration (%) (3)
  where Straw Total N content was calculated as:
(4) Straw Total N (kg N·ha^−1^) = Straw Yield (kg·ha^−1^) × Straw N concentration (%) (4)
(5) Total N at GS65 content was calculated as:
  GS65 Total N (kg N·ha^−1^) = Biomass (kg·ha^−1^) × N (%) (5)

Biomass was calculated, and the N concentration was analysed using the Kjeldhal procedure under all conditions [35]. 

### 4.6. Measurements Using Yara N-Tester^TM^

Yara N-Tester^TM^ (Yara International ASA, Oslo, Norway) is a chlorophyll meter that measures light transmitted by the plant leaf at two different wavelengths, 650 (red light) and 940 nm (near-infrared light). The instrument processes a digital reading calculated from the light transmitted by the plant leaf and light transmitted with no sample. 

The chlorophyll meter calculates a value (*M*) that is determined as follows:

(6) $$M=k\cdot \;log10\frac{Io\left(650\right)I\left(960\right)}{I\left(650\right)Io\left(960\right)}$$

where *Io* is the intensity of the incident monochromatic light and *I* is the intensity of the transmitted light. The K value is instrument dependent. 

Yara N-Tester^TM^ readings were taken for all treatments (Table 3) and GS65. The measurements were taken in the last fully developed leaf in the middle of the blade. Thirty main-stem flag leaves were measured at random along with the plots. A mean value was calculated for each plot. The acquired values were expressed as the relative chlorophyll content and were unitless. 

### 4.7. Statistical Analysis

The three factors that influence the GPC, yield, Yara N-Tester^TM^ at GS65, the N content in the post-anthesis period, and the grain total N content were the growing season, initial fertilisation treatment, and N rate at GS30, were analysed by analysis of variance (ANOVA) using ‘R 3.2.5’ software. To separate the means, Duncan’s test was used (*p* < 0.05) using the R package *agricolae*.

Coefficients of determination (*R*^2^) were calculated for the relationships between the Yara N-Tester^TM^ values at GS65 and GPC (%) for each growing season (2015, 2016, and 2017) using ‘R 3.2.5’ software. Otherwise, the Cate–Nelson procedure was performed to determine the accuracy of Yara N-Tester^TM^ values at GS65 to predict GPC (%). The Cate–Nelson procedure is used to divide data in two populations: One where a change in the *X* variables is likely to correspond to a change in the *Y* variable, and the other group where a change in the *X* is unlikely to correspond to a change in *Y*. 

## 5. Conclusions

The type of initial fertiliser did not affect the GPC values and chlorophyll meter readings at mid-anthesis. Generally, the higher the mineral N applied at stem elongation, the higher the chlorophyll meter readings and GPC values, because the GPC values are yield dependent. The chlorophyll meter readings at mid-anthesis in wheat might be helpful in estimating the GPC values under humid Mediterranean conditions only when the yields are below 8000 kg·ha^−1^. Yara N-Tester^TM^ readings at mid-anthesis should be higher than 700 in the wheat Cezanne variety to achieve the recommended level of GPC for high-quality bread-making flour (12.5%) at these yield levels. These results will allow farmers and cooperatives to make better decisions regarding late-nitrogen fertilisation and product sales, but it is necessary to adjust the values to the different varieties or cultivars. Future directions for wheat grain protein estimation should explore new fertilisation strategies including late N applications with granular mineral fertilisers or foliar applications but always avoiding the N rate increase.

## Figures and Tables

**Table 1:** Wheat grain protein content (GPC), grain yield (kg ha^−1^), and Yara N-Tester^TM^ readings at GS65 (mid-anthesis [6]) in the field experiment located in Arkaute in 2015, 2016, and 2017.

Initial Fertilisation	Treatment	2015			2016			2017		
		GPC (%)	Yield (kg ha^−1^)	N-Tester GS65	GPC (%)	Yield (kg N ha^−1^)	N-Tester GS65	GPC (%)	Yield (kg N ha^−1^)	N-Tester GS65
**Conventional**	**40 + 0N**	8.2 ± 2.4 BC	4942 ± 555 C	469 ± 47 C	8.3 ± 1.3	6083 ± 755 C	456 ± 65	8.4 ± 0.3 D	5239 ± 192 C a	478 ± 15 C
	**40 + 40N**	7.7 ± 0.3 C	7078 ± 912 B	530 ± 23 B	7.9± 1.3	8507 ± 203 B	474 ± 9	9.3 ± 0.5 C	5905 ± 488 B a	578 ± 18 B
	**40 + 80N**	8.6 ± 0.3 BC	8215 ± 548 A	585 ± 29 A	8.0 ± 1.0	9682 ± 357 A	539 ± 42	10.8 ± 0.3 B	6492 ± 450 AB a	604 ± 48 B
	**40 + 120N**	9.7 ± 0.8 AB	8230 ± 144 A	614 ± 12 A	9.5 ± 1.0	9933 ±630 A	551 ± 51	11.5 ± 0.9 B	6941 ± 202 A a	687 ± 3 A
	**40 + 160N**	10.4 ± 0.8 A	8688 ± 812 A	625 ± 9 A	9.5 ± 1.6	10554 ± 401 A	570 ± 57	12.9 ± 0.5 A	7095 ± 404 A a	710 ± 18 A
**Dairy slurry**	**DS + 0N**	8.1 ± 0.9 BC	4378 ± 145 C	489 ± 18 D	9.6 ± 2.1	5969 ± 525 C	453 ± 28 B	8.6 ± 0.3 E	3879 ± 168 C b	460 ± 11 C
	**DS + 40N**	7.7 ± 0.4 C	6271 ± 56 B	547 ± 31 C	7.5 ± 0.9	8431 ± 480 B	467 ± 26 B	9.6 ± 0.4 D	5081 ± 248 B b	570 ± 22 B
	**DS + 80N**	8.7 ± 0.2 BC	7762 ± 316 A	570 ±19 B	8.5 ± 1.8	10136 ± 560 A	538 ± 35 A	10.9 ± 0.4 C	5965 ± 322 A b	605 ± 46 B
	**DS + 120N**	10.3 ± 0.8 A	8275 ± 345 A	601 ± 17 B	9.2 ± 2.0	10221 ± 426 A	562 ± 17 A	12.2 ± 0.4 B	6056 ± 589 A b	661 ± 26 A
	**DS + 160N**	10.6 ± 0.9 A	8181 ± 961 A	644 ± 18 A	10.0 ± 3.3	10262 ± 373 A	610 ± 45 A	13.2 ± 0.6 A	6137 ± 104 A b	677 ± 8 A
**Sheep manure**	**SM + 0N**	7.4 ± 0.4C	4807 ± 588 C	503 ± 12 C	8.9 ± 1.6	6659 ± 801 D	452 ± 21 C	9.2 ± 0.4 E	3472 ± 196 D b	484 ± 5 C
	**SM + 40N**	7.7 ± 0.3 BC	6525 ± 261 B	521 ±28 C	10.0 ± 1.4	8803 ± 424 C	516 ± 33 B	10 ± 0.2 D	4704 ± 445 C b	562 ± 7 B
	**SM + 80N**	8.8 ± 0.3 BC	7966 ± 244 A	579 ± 20 B	7.7 ± 1.0	9518 ± 336BC	535 ± 39 B	10.8 ± 0.4 C	5287 ± 413 B b	604 ± 10 B
	**SM + 120N**	9.3 ± 1.7 B	8154 ± 368 A	623 ± 39 A	8.4 ± 0.8	10446 ± 681 AB	575 ± 6 A	12 ± 0.4 B	5537 ± 290 AB b	653 ± 44 A
	**SM + 160N**	10.9 ± 1.0 A	8525 ± 452 A	639 ± 17A	8.6 ± 0.8	10772 ± 726 A	601 ± 48 A	13.3 ± 0.3 A	5923 ± 314 A b	683 ± 11 A
**Control**	**0N**	7.5 ± 0.1	4119 ± 277	652 ± 43	7.7 ± 1.1	5243 ± 182	409 ± 72	8.51 ± 0.3	3348 ± 320	478 ± 15
**Overfert.**	**280N**	nd	nd	657 ± 24	8.6 ± 1.1	9375 ± 911	550 ± 6	13.1 ± 0.2	8020 ± 268	683 ± 11

Uppercase letters represent differences (*p* < 0.05) among different N rates applied at GS30 (X + 0N, X + 40N, X + 80N, X + 120N, X + 160N) for each initial fertilisation treatment in each growing season. Lowercase letters represent differences (*p* < 0.05) among different initial fertilisation treatments (conventional, dairy slurry, or sheep manure) for each N rate applied at GS30 in each growing season. The absence of uppercase or lowercase letters indicates that no significant differences (*p* > 0.05) were detected. *nd,* no data; ± *sd* (standard deviation); 0N and 280N were not included in ANOVA.

**Table 2:** Wheat crop total N content at harvest (kg N ha^−1^) and increase in the N content in the aerial part of the crop (kg N ha^−1^) during the post-anthesis period (from GS65 to harvest).

Treatment	2015		2016		2017	
	Grain Total N	Post-Anthesis N Increase	Grain Total N	Post-Anthesis N Increase	Grain Total N	Post-Anthesis N Increase
40N + 0N	66 ± 17 D	10 ± 1	84 ± 18 D	31 ± 11	70 ± 3 E	30 ± 6 a
40N + 40N	90 ± 14 C	32 ± 9	113 ± 17 C	51 ± 18	88 ± 11 D	18 ± 5
40N + 80N	116 ± 12 B	21 ± 6	130 ± 19 B	31 ± 2	112 ± 6 C	37 ± 11
40N + 120N	132 ± 14 AB	10 ± 5	157 ± 17 AB	59 ± 22	128 ± 13 B	11 ± 6
40N + 160N	149 ± 20 A	20 ± 4	168 ± 33 A	44 ± 18	146 ± 6 A	28 ± 9
DS + 0N	59 ± 8 D	15 ± 5	96 ± 24 C	53 ± 26	53 ± 4 E	13 ± 5 b
SM + 0N	58 ± 5 D	6 ± 2	101 ± 29 C	43 ± 14	51 ± 3 E	19 ± 3 b
0N	51 ± 4	17 ± 7	63 ± 17	24 ± 8	46 ± 4	11

Uppercase letters represent differences among different N rates applied at GS30 (40N + 0N, 40N + 40N, 40N + 80N, 40N + 120N, 40N + 160N) for each initial fertilisation treatment. Lowercase letters represent differences (*p* < 0.05) among different initial fertilisation treatments (conventional, dairy slurry, or sheep manure) for the 0 kg N·ha^−1^ rate applied at GS30 in each growing season. The absence of uppercase or lowercase letters indicates that no significant differences (*p* > 0.05) were detected. ± *sd* (standard deviation);. 0N was not included in ANOVA. DS, dairy slurry; SM, sheep manure.

**Table 3:** N application rates and timing in three initial fertilisation treatments and three growing seasons (2015, 2016, and 2017) in the field experiment located in Arkaute. Control (0 N) and overfertilised (280 N). GS21, beginning of tillering; GS30, stem elongation [6].

Initial Fertilisation	Topdressing at GS21 (kg N·ha^−1^)	Topdressing at GS30 (kg N·ha^−1^)	Treatment Identification
Conventional [-]	40	0	40N + 0N
		40	40N + 40N
		80	40N + 80N
		120	40N + 120N
		160	40N + 160N
Dairy Slurry (DS) [40 t·ha^−1^]	--	0	DS + 0N
		40	DS + 40N
		80	DS + 80N
		120	DS + 120N
		160	DS + 160N
Sheep manure (SM) [40 t·ha^−1^]	--	0	SM + 0N
		40	SM + 40N
		80	SM + 80N
		120	SM + 120N
		160	SM + 160N
Control [-]	--	--	0N
Overfertilised [-]	80	200	280N

## Data Availability

The data presented in this study are available on request from the corresponding author.

## Supplementary Materials

The following are available online at https://www.mdpi.com/2223-7747/10/2/374/s1. Table S1: Duncan’s test (*p* < 0.05) results of the differences in the GPC values (%), yield (kg ha^−1^), and Yara N-Tester values at mid-flowering (GS65, [6]) among different wheat growing seasons (2015, 2016 and 2017) for each initial ferti-lisation treatment (conventional, dairy slurry and sheep manure) and each N dose (0, 40, 80, 120 and 160 kg N ha^−1^) applied at stem elongation (GS30, [6]) in the field study at Arkaute. Table S2: Duncan’s test (*p* < 0.05) results of the differences in the grain total nitrogen (kg N ha^−1^) and post-anthesis ni-trogen increase (kg N ha^−1^) among different wheat growing seasons (2015, 2016 and 2017) for each treatment in the field study at Arkaute. Table S3: Total rainfall (mm), cumulative growing degree days GDD (°C) and days elapsed between wheat growing stages [6] in three growing seasons (2015, 2016 and 2017) in the field study at Arkaute.
