# Supplementary material for: Wheat Grain Protein Content under Mediterranean Conditions Measured with Chlorophyll Meter
Source: Plants (Basel). 2021 Feb 15;10(2):374. doi: 10.3390/plants10020374 (PMC7919282; doi:10.3390/plants10020374)
Supplement: Supplementary file 1 [file plants-10-00374-s001.pdf]

plants

MDPI

Supplementary Materials:

Table S1. Duncan's test ($p<0.05$) results of the differences in the GPC values (%), yield (kg ha^{-1}), and Yara N-Tester values at mid-flowering (GS65, [6]) among different wheat growing seasons (2015, 2016 and 2017) for each initial fertilisation treatment (conventional, dairy slurry and sheep manure) and each N dose (0, 40, 80, 120 and 160 kg N ha^{-1}) applied at stem elongation (GS30, [6]) in the field study at Arkaute.

Initial fertilisation	Treatment	GPC (%)			Yield (kg N ha^{-1})			N-Tester GS65		
		2015	2016	2017	2015	2016	2017	2015	2016	2017
Conventional	40 + 0N		*ns*		B	A	AB		*ns*	
	40 + 40N		*ns*		B	A	C	B	C	A
	40 + 80N	B	B	A	B	A	C	AB	B	A
	40 + 120N	B	B	A	B	A	C	B	C	A
	40 + 160N	B	B	A	B	A	C	B	C	A
Dairy slurry	DS + 0N		*ns*		B	A	B	A	B	B
	DS + 40N	B	B	A	B	A	B	A	B	A
	DS + 80N	B	B	A	B	A	B	AB	B	A
	DS + 120N	AB	B	A	B	A	B	B	C	A
	DS + 160N		*ns*		B	A	B	AB	B	A
Sheep manure	SM + 0N	B	A	A	B	A	B	A	B	A
	SM + 40N	B	B	A	B	A	B	B	B	A
	SM + 80N	B	C	A	B	A	B	A	B	A
	SM + 120N	B	B	A	B	A	B	A	B	A
	SM + 160N	B	C	A	B	A	B	AB	B	A

Different uppercase letters represent differences among wheat growing seasons (2015, 2016, 2017) for each initial fertilisation treatment (conventional, dairy slurry, sheep manure) and each N rate applied at GS30 (0, 40, 80, 120, 160 kg N ha^{-1}). *ns,* not significant.

Table S2. Duncan's test ($p<0.05$) results of the differences in the grain total nitrogen (kg N ha^{-1}) and post-anthesis nitrogen increase (kg N ha^{-1}) among different wheat growing seasons (2015, 2016 and 2017) for each treatment in the field study at Arkaute.

Treatment	Grain Total N			Post-anthesis N increase		
	2015	2016	2017	2015	2016	2017
40N+0N		*ns*		B	A	A
40N+40N	B	A	B		*ns*	
40N+80N		*ns*			*ns*	
40N+120N		*ns*		B	A	B
40N+160N		*ns*		B	A	AB
DS+0N	B	A	B	B	A	B
SM+0N	B	A	C	C	A	B

Different uppercase letters represent differences among wheat growing seasons (2015, 2016, 2017) for each treatment. *ns*, not significant.

Table S3. Total rainfall (mm), cumulative growing degree days GDD (ºC) and days elapsed between wheat growing stages [6] in three growing seasons (2015, 2016 and 2017) in the field study at Arkaute.

Growing season	Growing stage	Total rainfall (mm)	Cumulative GDD (℃)$^{+}$	Days elapsed
2015	Sowing (24/11) - GS21 (09/03)	521.2	556	106
	GS21 (09/03) - GS30 (04/04)	94.6	788	30
	GS30 (04/04) - GS32 (29/04)	43.2	1048	21
	GS32 (29/04) - GS37 (11/05)	6	1235	12
	GS37 (11/05) - GS65 (28/05)	13.8	1432	17
	GS65 (28/05) - Harvest (21/07)	55.5	2462	54
2016	Sowing (06/11) - GS21 (19/01)	168.1	611	74
	GS21 (19/01) - GS30 (17/03)	296.1	994	56
	GS30 (17/03) - GS32 (30/03)	16.5	1118	13
	GS32 (30/03) - GS37 (06/04)	24.3	1126	7
	GS37 (06/04) - GS65 (25/05)	71.2	1721	49
	GS65 (25/05) - Harvest (02/08)	70.5	3005	69
2017	Sowing (18/11) - GS21 (02/03)	271.3	635	105
	GS21 (02/03) - GS30 (06/04)	57.6	976	35
	GS30 (06/04) - GS32 (12/04)	0	1078	6
	GS32 (12/04) - GS37 (25/04)	0	1218	13
	GS37 (25/04) - GS65 (30/05)	82.4	1718	35
	GS65 (30/05) - Harvest (02/08)	114.3	2980	63

$^{+}$The cumulative GDD was calculated with 5 °C as the baseline.
